# Allelic phenotype prediction of phenylketonuria based on the machine learning method

**DOI:** 10.1186/s40246-023-00481-9

**Published:** 2023-03-31

**Authors:** Yang Fang, Jinshuang Gao, Yaqing Guo, Xiaole Li, Enwu Yuan, Erfeng Yuan, Liying Song, Qianqian Shi, Haiyang Yu, Dehua Zhao, Linlin Zhang

**Affiliations:** 1 Department of Laboratory Medicine, Third Affiliated Hospital of Zhengzhou University, 7 Kangfu Qian Street, Zhengzhou, 450052 Henan People’s Republic of China; 2 Neonatal Screening Center, The Third Affiliated Hospital of Zhengzhou University, 7 Kangfu Qian Street, Zhengzhou, 450052 Henan People’s Republic of China

**Keywords:** PKU, Case database, Genotype–phenotype prediction, Machine learning, PKU network

## Abstract

**Background:**

Phenylketonuria (PKU) is caused by mutations in the phenylalanine hydroxylase (PAH) gene. Our study aimed to predict the phenotype using the allelic genotype.

**Methods:**

A total of 1291 PKU patients with 623 various variants were used as the training dataset for predicting allelic phenotypes. We designed a common machine learning framework to predict allelic genotypes associated with the phenotype.

**Results:**

We identified 235 different mutations and 623 various allelic genotypes. The features extracted from the structure of mutations and graph properties of the PKU network to predict the phenotype of PKU were named PPML (PKU phenotype predicted by machine learning). The phenotype of PKU was classified into three different categories: classical PKU (cPKU), mild PKU (mPKU) and mild hyperphenylalaninemia (MHP). Three hub nodes (c.728G>A for cPKU, c.721 for mPKU and c.158G>A for HPA) were used as each classification center, and 5 node attributes were extracted from the network graph for machine learning training features. The area under the ROC curve was AUC = 0.832 for cPKU, AUC = 0.678 for mPKU and AUC = 0.874 for MHP. This suggests that PPML is a powerful method to predict allelic phenotypes in PKU and can be used for genetic counseling of PKU families.

**Conclusions:**

The web version of PPML predicts PKU allele classification supported by applicable real cases and prediction results. It is an online database that can be used for PKU phenotype prediction http://www.bioinfogenetics.info/PPML/.

**Supplementary Information:**

The online version contains supplementary material available at 10.1186/s40246-023-00481-9.

## Background

Phenylketonuria (PKU, OMIM# 261600) is a common autosomal recessive inherited metabolic disease with an inborn error of phenylalanine (Phe) metabolism, which is caused by pathogenetic variants in the phenylalanine hydroxylase (PAH) gene [1]. In the BIOPKU database, approximately 73% of genotypes were compound heterozygous, 27% were homozygous, and 55% of genotypes occurred in only a single individual [2]. Untreated PKU can generally lead to severe irreversible damage, such as intellectual disability, seizures, behavioral problems and mental disorders, and it may also result in a musty smell and lighter skin [3, 4]. A baby born to a mother who has poorly treated PKU may have heart problems and low birth weight [5, 6]. The danger and complexity of PKU make it a challenging task to understand and explore the relationship between PAH mutant genes and phenotypes.

It is difficult for patients with phenylalanine hydroxylase (PAH) gene variants that result in elevated concentrations of Phe in the blood to convert phenylalanine to tyrosine [7]. The severity of the disorder varies between patients depending on the blood Phe level at the time of diagnosis or dietary Phe tolerance. In earlier studies, the phenotypes of PAH deficiency were classified into four categories: classic PKU (cPKU), moderate PKU (moPKU), mild PKU (miPKU) and mild hyperphenylalaninemia (MHP) with pretreatment blood Phe concentrations of > 1200 μmol/L, 900–1200 μmol/L, 600–900 μmol/L, 360–600 and 120–360 μmol/L, respectively [8, 9]. Recently, more research has combined moderate PKU and mild PKU, dividing the phenotype into three categories [2, 3, 8, 10]. The prevalence of PKU varies among ethnicities and geographic areas. The global prevalence of PKU was estimated to be 1:23,930 newborns, and the prevalence of PKU in China was 1:15,924 [2]. The phenotype prevalence of the three categories in the chain was 62% cPKU, 25% mPKU and 35% MHP [1].

Genotype–phenotype plays an important role in PKU patients that can guide the treatment strategy and can also predict the prognosis. Several methods of genotype-based phenotype prediction have been reported, such as the arbitrary values method (AV) [9] and allelic phenotype values method (APV) [10]. The AV method classified the PKU phenotype into four arbitrary phenotype categories (cPKU, moderate PKU, mPKU and MHP) based on 297 functionally hemizygous patients and 105 PAH mutations. The APV method used 9336 PKU patients with 2589 different genotypes carrying 588 variants to investigate the PKU phenotype. This study identified 251 0-variant encoding inactive PAH and 88 variants in PAH functional hemizygous patients and classified the PKU phenotype into three categories (cPKU, mPKU and HPA). Both methods share a common feature: They score genotypes based on hemizygous patients, and then, the scores of the two combined alleles are summed to predict the final PKU phenotype. However, this approach has the inherent limitation that sufficient information on hemizygous patient mutations must be available for predicting PKU phenotypes; otherwise, the prediction is difficult to perform. Therefore, we propose a method to predict PKU phenotypes for arbitrary allele combinations without using the status of hemizygous patients.

In this study, we provide a new method to predict genotype–phenotype based on the machine learning method. A total of 1291 PKU patients were used as the training data for classifying the PKU phenotype into three categories: cPKU, mPKU and HPA. The features were extracted from the information of nucleotide mutations and amino acid change information, as well as the property of the allelic mutation linkage graph, for training with machine learning classification models. PKU phenotype prediction based on intrinsic information of PAH gene mutation loci can avoid the constraint of obtaining sufficient information on hemizygous patients, thus allowing the prediction of arbitrary allele combinations. Furthermore, this work improves the accuracy of PKU phenotype prediction by the ML method.

## Materials and methods

### Participants

A total of 1291 PKU cases over 14 years were collected in this study; 769 PKU patients were recruited at the Third Affiliated Hospital of Zhengzhou University between January 2016 and January 2022, and the remaining 522 PKU patients were collected from a study by Liu et al. [11], in which PKU patients were recruited from January 2008 to January 2016. We excluded patients with BH4 cofactor deficiency in this work. In this study, all subjects or guardians provided signed informed consent. This study was approved by the Medical Ethics Committee of the Third Affiliated Hospital of Zhengzhou University and was performed according to the principles of the Declaration of Helsinki.

### Training data

In this study, we classified all PKU cases into three metabolic phenotype groups, classical PKU (cPKU, pretreatment blood Phe > 1200 µmol/L), mild PKU (mPKU, pretreatment blood Phe 600–1200 µmol/L) and mild hyperphenylalaninemia (MHP, pretreatment blood Phe 120–600 µmol/L). Of the 1291 PKU cases, 638 patients were classified as cPKU, 295 as mPKU and 358 as MHP. We then obtained three group training datasets for cPKU, mPKU and MHP. For cPKU, the training dataset contained 638 positive alleles and 653 negative alleles; the training dataset for mPKU contained 295 positive alleles and 996 negative alleles, and the MHP training dataset contained 358 positive alleles and 993 negative alleles.

### Structure feature

We designed a new method to predict the phenotype of PKU based on allelic genotype by the ML method. The most critical part of this approach is extracting the corresponding feature values based on the obtained information for the training of machine learning models. First, we extracted the feature based on the mutation information, which we call the structure feature. We encoded the nucleic acid mutations, amino acids and exon/intron information with a different real number, and then, we extracted 7 dimensions for each mutation. For the allele genes, the dimension is 7 + 7 = 14. The detailed method about encodes structure feature can be found in Additional file 1: Supplementary note.

### Graph features

We extracted 8 feature values from the network graph of the alleles. In the network, we identified three hub nodes by Cytoscape software [12]. We extracted 3 distance features that calculated the minimum distances to three hub nodes, including the minimum distance to the cPKU hub node, minimum distance to the mPKU hub node and minimum distance to the MHP hub node. Five node attributes were also calculated, including the degree measures and the number of direct interacting partners of this mutation. PageRank is the famous algorithm used by Google Search to rank web pages. Here, we used this algorithm to estimate how important the mutation was in the network. Edge betweenness measured how often the mutation was involved in the shortest paths in the network. By the network graph, we extracted 8 features from each mutation. The total dimension of alleles is 8 + 8 = 16. The detailed method for encodes graph feature can be found in Additional file 1.

### Predicting allelic phenotype

Different methods have been proposed to classify phenotypes. First, the mutations were assigned to four phenotype categories (classic PKU, moderate PKU, mild PKU and MHP) proposed by Guldberg et al. [9]. In the most recent studies, the phenotype of PKU has been classified into three different categories by merging moderate PKU and mild PKU into one classification (classical PKU, mild PKU and MHP) [2, 10]. In this study, we generated an ML training dataset with three phenotype categories. The random forest (RF) method was used to predict allelic phenotypes that were implemented by the Python-based library scikit-learn [13]. To conduct a stringent performance assessment, tenfold cross-validation tests were carried out. We chose the receiver operating characteristic curve (ROC curve) and the area under the ROC curve (AUC) to assess the performance of our models. The formulas to calculate the true positive rate (TPR), false positive rate (FPR) and specificity (TNR) are as follows:

$$\mathrm{TPR}=\mathrm{sensitivity}=\frac{\mathrm{TP}}{\mathrm{TP}+\mathrm{FN}}$$

$$\mathrm{FPR}=\frac{\mathrm{FP}}{\mathrm{FP}+\mathrm{TN}}$$

$$\mathrm{TNR}= \mathrm{specificity}=\frac{\mathrm{TN}}{\mathrm{TN}+\mathrm{FP}}$$

## Results

### Distribution of mutations and phenotypes

**Fig. 1:**
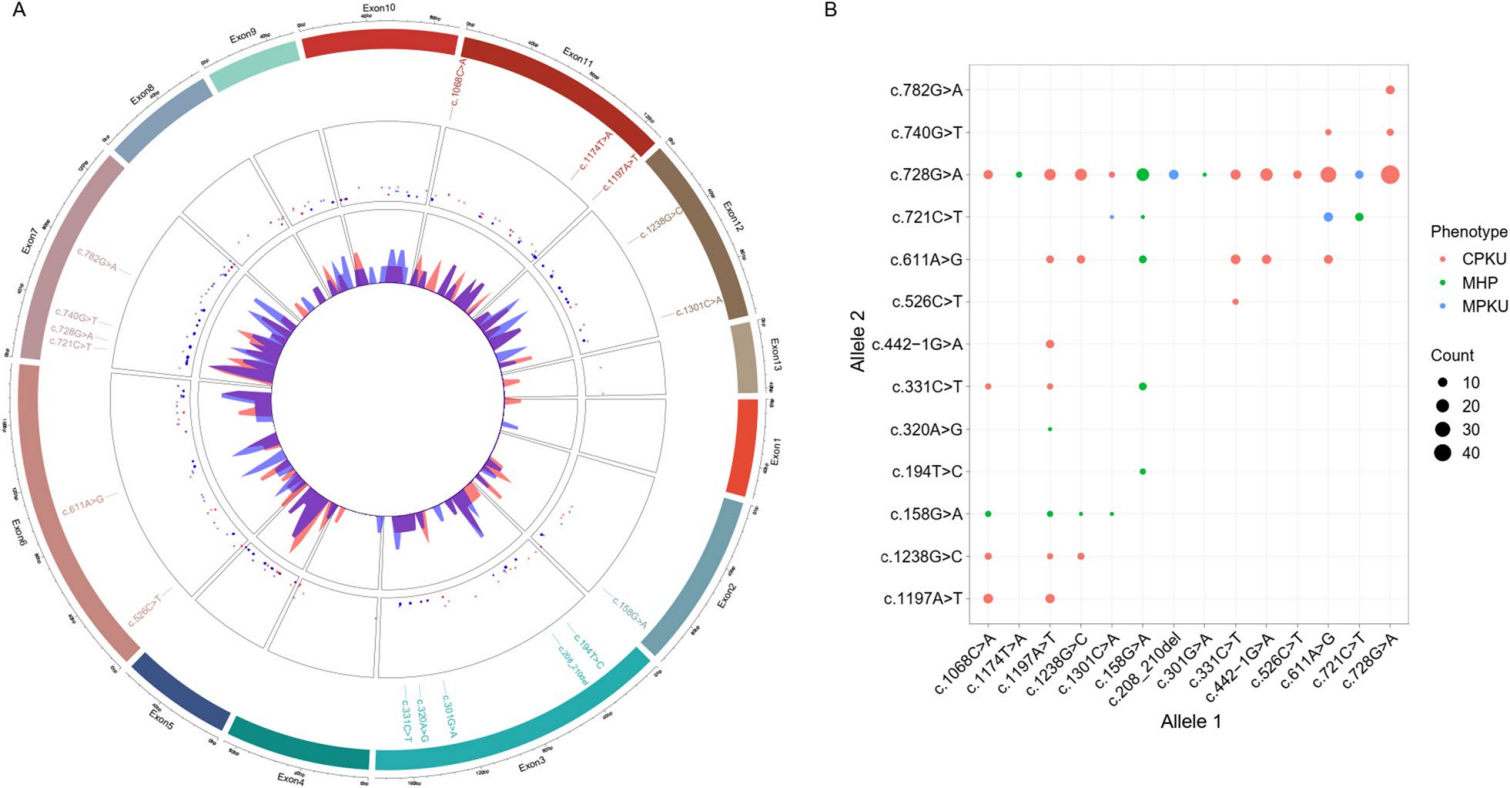
Distribution of mutations for allele genes. **A** Location information of mutations in the PAH gene. **B** The allele genotype is associated with the PKU phenotype

A total of 1291 unrelated patients were investigated. A total of 235 different mutations were discovered, and 623 various allelic genotypes were identified. Figure 1A shows the distribution of mutations for allele genes. What stands out in the distribution is that the mutations covered all exonic regions of the PAH gene, except for Exon 1, Exon 4 and Exon 13, which were less distributed. The highest frequency of mutations was distributed in exon 2 (c.158G>A); exon 3 (c.194T>C, c.208_210del, c.301G>A, c.320A>G, c.311C>T); exon 6 (c.526C>T, c.611A>G); exon 7 (c.721C>T, c.728G>A, c.740G>A, c.782G>A); exon 11 (c.1068C>A, c.1174T>A, c.1197A>T); and exon 12 (c.1238G>C, c.1301C>A). Three different phenotype categories were classified according to the pretreatment plasma phenylalanine levels: cPKU (classic PKU), mPKU (mild PKU) and MHP (mild hyperphenylalaninemia). The 623 different mutations (compound heterozygous and homozygous) are shown in Additional file 2: Table S1. The high frequency of different PAH mutations combined into 43 different genotypes is shown in Fig. 1B. The mutations c.728G>A, c.611A>G, c.1.068C>A and c.1197A>T are the four most important mutations leading to the cPKU phenotype. What is striking about the cPKU phenotype is that the four mutations combined with c.158G>A result in MHP phenotypes. Details about the cPKU phenotype are shown in Additional file 3: Fig. S1. Moreover, c.158G>A combined with any mutation will lead to the MHP phenotype. This suggests that c.158G>A is a significant mutation that leads to the MHP phenotype. What is interesting about the combination of these mutations is the mPKU phenotype. For example, the mutation c.721C>T combined with important cPKU mutations (such as c.728G>A and c.611A>G) will lead to mPKU, but when c.721C>T is combined with itself or an important HPA mutation (c.158G>A), it results in MHP (Fig. 1B). The allelic genotypes associated with mPKU and MHP are shown in Additional file 4: Fig. S2 and Additional file 5: Fig. S3. The above results show the effect of different allele mutation combinations on phenotype and support the AV method proposed by Guldberg et al., which predicts phenotype by combining different genotypes [9]. Since the combination of alleles with different mutations (compound heterozygous and homozygous) leading to different PKU phenotypes is very complex, it becomes very difficult to predict phenotypes by simple allele combinations. Therefore, we proposed a new method to predict the allelic phenotype of PKU by machine learning.

### Predicting the PKU phenotype model

**Fig. 2:**
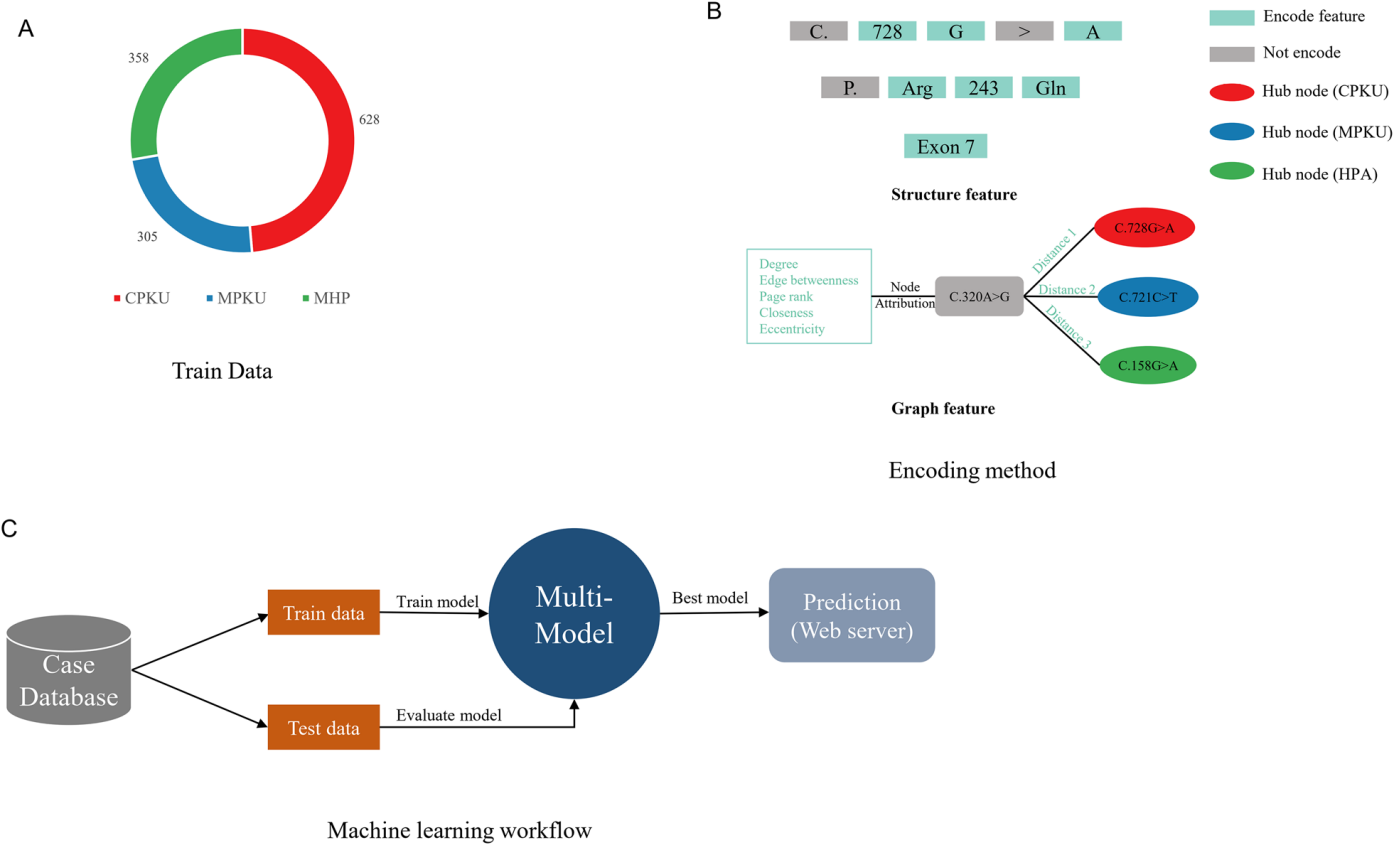
Scheme of the framework of PPML. **A** The PKU phenotype is classified in the training dataset. **B** Encoding the features extracted from the structure and graph method. **C** Workflow of PPML to predict the PKU phenotype

Our research aimed to design a generic framework for predicting the allelic phenotype in the PAH gene that causes PKU. We designed a new method named PPML (**P**KU** p**henotype predicted by **m**achine** l**earning) that extracts the training features from mutation information to predict the allelic phenotype. Figure 2 shows the framework of PPML. A total of 1291 PKU cases used as a training dataset in this study contained 628 cPKU, 305 mPKU and 358 MHP patients (Fig. 2A). In the PPML framework, the training features are extracted in two ways: One way is based on the structural information of the mutation, and the other is based on the connectivity network graph composed of the mutations. As shown in Fig. 2B, the encoding method for structural information contains a nucleic acid mutation, amino acid change and exon/intron information in the PAH gene. We linked all the allelic mutations together to form a network graph. Each mutation as a node in the network graph is connected except for 12 mutations (Additional file 6: Fig. S4). We suspect that if enough case data are collected, then all mutations in the graph will form a linked network. This allows us to use the basic properties of graphs to encode feature values for ML training. Here, a total of 5 node attributes were extracted from the network graph, including degree, edge betweenness, page rank, closeness and eccentricity. Moreover, we identified three hub nodes (c.728G>A for cPKU, c.721C>T for mPKU and c.158G>A for HPA) as each classification center by Cytoscape software [12] (Additional file 7: Fig. S5). The distance to each hub node is another three features. Therefore, a total of 8 features were extracted from the allelic mutation network graph. The machine learning workflow is shown in Fig. 2C. The collected PKU patient cases were split into training data and test data for machine learning. A multimodel classification method was used in this framework, and the best model was chosen to predict PKU. Finally, the prediction results were reported by the webserver.

### The performance of PPML

**Fig. 3:**
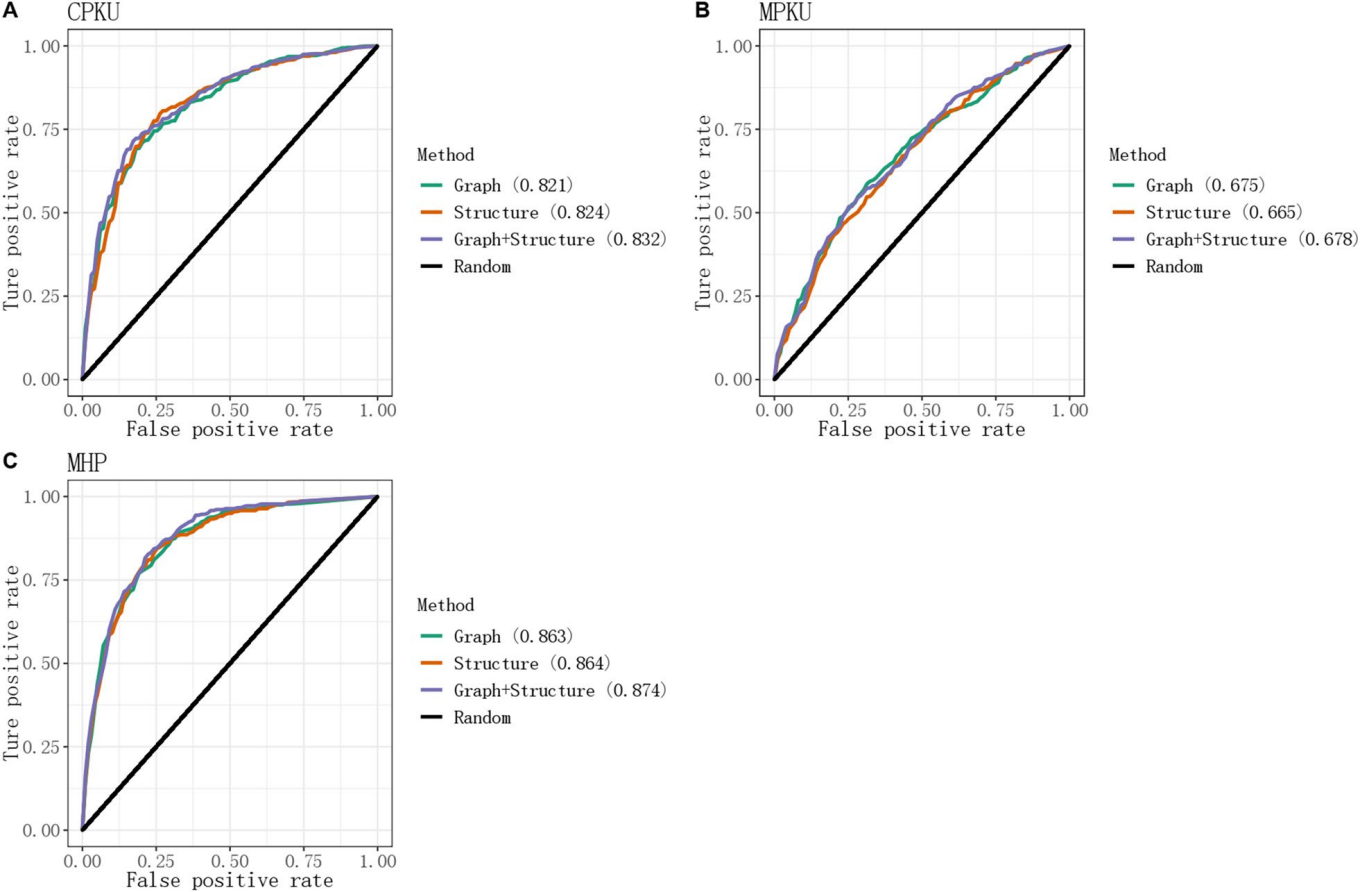
Performance of the structure and graph method for phenotype categories. **A** Performance in the cPKU training dataset. **B** Performance in the mPKU training dataset. **C** Performance in the MHP training dataset

In this study, random forest (RF) is the classified model for predicting the phenotype of PKU. The receiver operating characteristic curve (ROC curve) and the area under the ROC curve (AUC) were used to assess the performance of the three different categories. The performance of the various training features is shown in Fig. 3. The best performance is the combined graph and structure feature (AUC = 0.832 in the cPKU test dataset, AUC = 0.678 in the mPKU test dataset and AUC = 0.874 in the MHP test dataset). The structure-based approach was better than the graph-based approach in the three different test datasets, except for mPKU with AUC = 0.675 (graph method) and AUC = 0.665 (structure method). In the three different datasets of test results, we can see that the structure-based and graph-based approaches perform essentially equally, and the performance of the combined graph-based and structure-based approaches was best in each test dataset. To avoid overfitting and ensure the generalizability of our method, we split our dataset into two subsets, with 80% of the data used for training and tenfold cross-validation, and the remaining 20% used as an independent validation set. The result of the RF method using the train dataset and the independent validation set are shown in Additional file 8: Table S2 and Additional file 9: Table S3, respectively. We compared the RF method with the other four classification methods (AdaBoost, Bernoulli, gradient boosting and K-neighbors method) using the cPKU training dataset (Additional file 10: Figure S6). The AUC results in this test showed that the RF method had the best performance with AUC = 0.832. The AUC performance by AdaBoost and gradient boosting classified methods was more than 0.8. Bernoulli and K-neighbors classified methods were poor performers, with AUC values of 0.511 and 0.685, respectively.

### Prediction of PKU phenotype web server

**Fig. 4:**
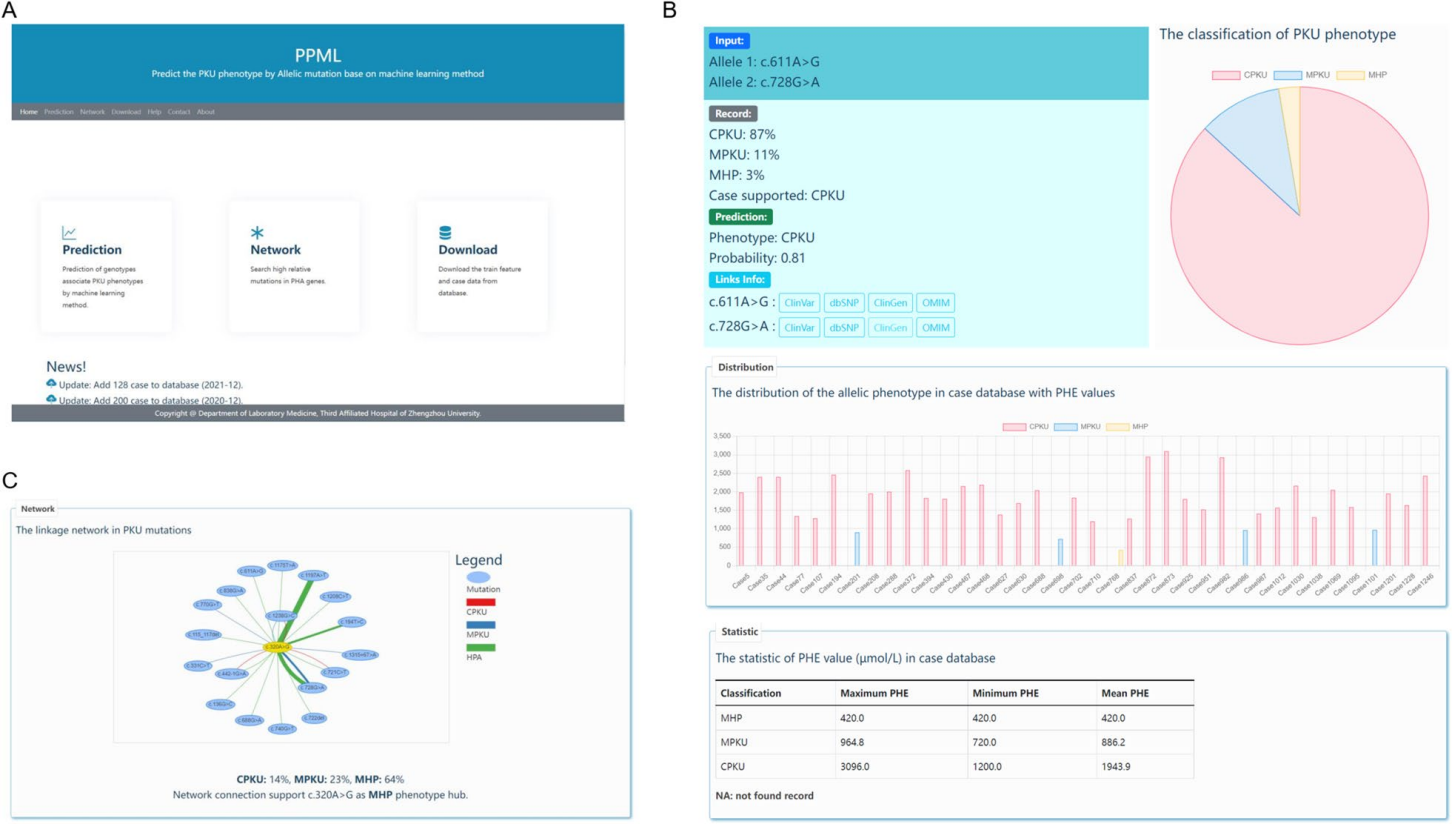
PPML web server for predicting the PKU phenotype. **A** Overview of the PPML web server. **B** Prediction of the PKU phenotype. **C** Mutations relative to the network with the PKU phenotype

We built an easy-to-use PKU phenotype prediction web server based on the collected 14 years of PKU patient data and the PPML common prediction framework (Fig. 4). Figure 4A shows an overview of the PPML database. The PPML database provided the prediction entry for predicting the PKU phenotype based on any combination of mutations in PAH genes; the network entry for searching the mutation's highly relative allele mutations and calculating the hub phenotype mutation for cPKU, mPKU and HPA; and download entry for retrieving the training dataset and case database. The predicted PKU phenotype, shown in Fig. 4B, contains the predicted phenotype and the probability. The combined mutations reported in the case database supported the PKU phenotype. The blood PHE value contains maximum PHE, minimum PHE and mean PHE in each classification. The highly relative allele mutations in the PPML case database were reported by a linkage network graph, and the mutation as a hub phenotype node was calculated (the phenotype classified as cPKU, mPKU and HPA) (Fig. 4C).

## Discussion

Since the activity of PAH, and thus the metabolic phenotype of PKU, is determined by genotype, the evidence of genotype–phenotype correlation is growing [14–17]. Over the past 20 years, many methods have been proposed for predicting PKU genotype–phenotype associations, and they have been widely used. With the power of large mutational databases, several methods have been proposed to predict the relationship between genotype and phenotype in PKU. For example, the AV [9] and APV [10] methods are based on the status of hemizygous patients compared to the allelic phenotype of PKU. Sarah et al. used APV methods to explore the genotype–phenotype correlation in phenylketonuria using data from locus-specific and genotype databases [18]. Tianwen et al. [19] and Santos et al. [20] analyzed genotype–phenotype correlation and estimated the damage caused to PAH by various missense mutations by the AV method and pairwise correlation analysis. The AV and APV methods for PKU genotype–phenotype inference require a large hemizygous patient database for support and simple allele combinations for phenotype prediction. The ML-based framework proposed in this study for genotype–phenotype inference does not require the support of a hemizygous patient database, only a partial training dataset is needed to predict the phenotype, and more databases can be added to make the prediction more accurate. On the other hand, Pey et al. provided an experimental framework to explore the severity relating genotype to phenotype of the mutations in PKU [15]. This method inferred PKU genotype–phenotype prediction by an experimental approach, which is time-consuming and labor-intensive. This study is based on a machine learning approach for PKU phenotype–genotype prediction, which can achieve rapid and large-scale inference.

Locus-specific databases play an important role in understanding the nature, prevalence and impact of PAH deficiency [21, 22]. The locus-specific databases PAHvdb, ClinVar, HGMD and LOVD were searched for PAH variant information. However, only PAHvdb explored the genotype–phenotype for PKU by linking to the BIOPKU database. Our research provided a new database for predicting the phenotype in PKU based on the ML method. It is essential to establish more databases for PKU genotype–phenotype prediction. Our research built the PPML database, which contains genotypes and clinical phenotypes of more than 1000 patients with PKU. In the PPML, 49% of patients had the classical phenotype and 24% had a mild phenotype, with the remainder having mild HPA. We found that if the amount of PKU data in the database is large enough, any two alleles can be linked to form a linkage graph (Figure S4). The network of alleles and the edges weighted by the count of PKU patients in the PPML database can be created. The prediction model of PPML explored the PKU phenotype by machine learning methods and clinical patient cases. PPML is a general framework that can be applied to predict PKU phenotypes in various populations. However, the prevalence of PKU is not uniform depending on the region. The prevalence of PKU varies substantially among ethnicities and between different geographic regions worldwide [1]. Here, our training dataset is only based on cases in central China, so the prediction results are more applicable to central China. In the future, we hope that this database can support more population studies with different PKU prevalence in different regions.

PAH is an iron-containing monooxygenase enzyme that catalyzes the hydroxylation of phenylalanine to form tyrosine [23]. This reaction requires molecular oxygen and BH4 as a cofactor. In this study, we excluded the BH4 responsiveness of patients from the training dataset because the relation between genotype and BH4 responsiveness is complex [14]. Although approximately 1–2% of cases of hyperphenylalaninemia are based on mutations in genes coding for enzymes involved in BH4 biosynthesis or regeneration [24, 25], some patients with defects in BH4 biosynthesis (such as Segawa disease and sepiapterin reductase deficiency) present without hyperphenylalaninemia [26, 27]. Our research focuses on genotypes based on PAH gene mutations for phenotype prediction in phenylketonuria. Therefore, this method does not apply to the prediction of the PKU phenotype due to BH4 responsiveness. We hope this method provides great help in being able to infer phenotypes from genotypes in clinical diagnosis.

## Supplementary Information

**Additional file 1.** Supplementary note. Encoding methods for structure and graph feature.

**Additional file 2: Table S1.** Allele mutations associated with PKU phenotypes.

**Additional file 3: Fig. S1.** Distribution of allelic genotype associated with cPKU.

**Additional file 4: Fig. S2.** Distribution of allelic genotype associated with mPKU.

**Additional file 5: Fig. S3.** Distribution of allelic genotype associated with HPA.

**Additional file 6: Fig. S4.** Connectivity of allelic mutations in the PAH gene.

**Additional file 7: Fig. S5.** Hub nodes for cPKU, mPKU and MPH.

**Additional file 8: Table S2.** Tenfold cross-validation on the training data, with a ratio of 8:2 for training and testing, respectively.

**Additional file 9: Table S3.** Independent validation by 20% of collection data.

**Additional file 10.** **Figure S6.** Compared the RF method with other classification methods.

## Data Availability

The machine learning model created during this study is available at http://www.bioinfogenetics.info/PPML/. The dataset used for machine learning training is provided on the website.

## Abbreviations

PKU: Phenylketonuria
Phe: Phenylalanine
cPKU: Classic PKU
moPKU: Moderate PKU
miPKU: Mild PKU
MHP: Mild hyperphenylalaninemia
AV: Arbitrary values method
APV: Allelic phenotype values method
ROC: Receiver operating characteristic curve
TPR: True positive rate
FPR: False positive rate
RF: Random forest
AUC: Area under the ROC curve
