# Supplementary material for: Allelic phenotype prediction of phenylketonuria based on the machine learning method
Source: Hum Genomics. 2023 Mar 31;17:34. doi: 10.1186/s40246-023-00481-9 (PMC10064562; doi:10.1186/s40246-023-00481-9)
Supplement: Supplementary file 1 — Additional file 1. Supplementary note. Encoding methods for structure and graph feature. [file 40246_2023_481_MOESM1_ESM.docx]

**Supplementary note for**

**The allelic phenotype prediction of phenylketonuria based on the machine learning method**

**Yang Fang^1*^, Jinshuang Gao^1^, Yaqing Guo^1^, Xiaole Li^2^, Enwu Yuan^1^, Erfeng Yuan^1^, Liying Song^1^, Qianqian Shi^1^, Haiyang Yu^1^, Dehua Zhao^2^, Linlin Zhang^1*^**

**Supplementary Note 1**

**Encode structure feature method**

We encode the nucleic acids mutations to real numbers ranging from 1 to 4, amino acids to real numbers ranging from 1 to 21 and Exon/Intron information to the position. The detailed encode framework and core of code about encode methods are as follow:


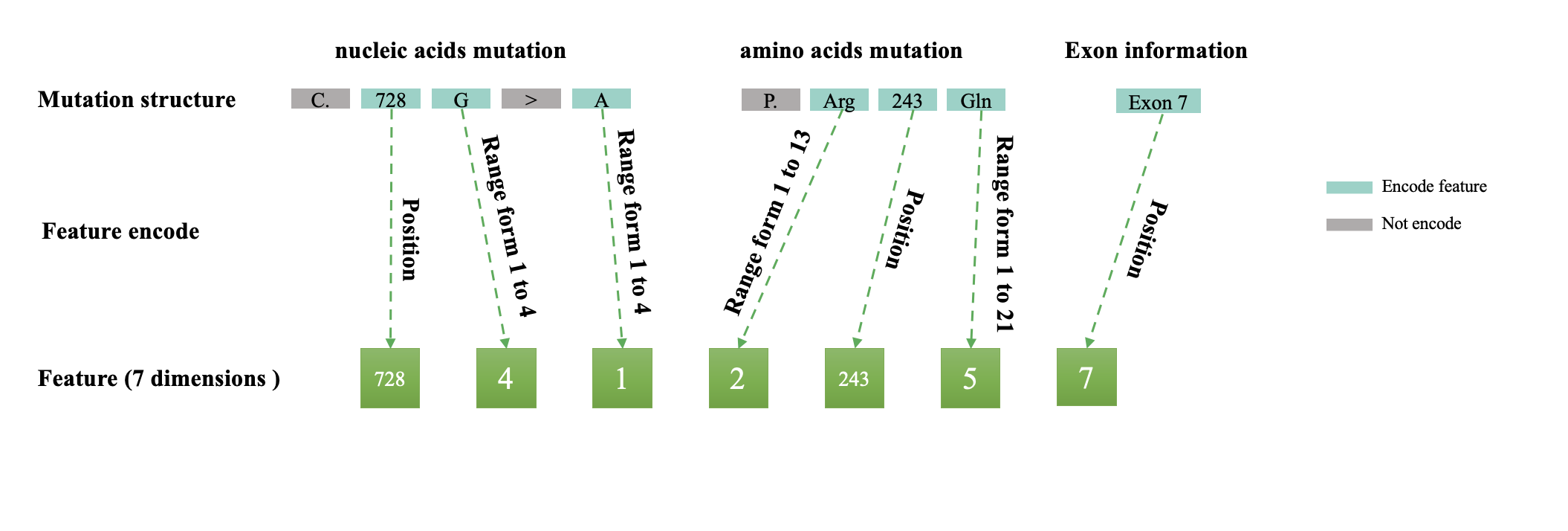


**Code Snippet**

c_feature = {"A": 1,
 "T": 2,
 "C": 3,
 "G": 4}

aa_feature = {"ala": 1, "arg": 2, "asn": 3, "asp": 4, "cys": 5,
 "gln": 6, "glu": 7, "gly": 8, "his": 9, "ile": 10,
 "leu": 11, "lys": 12, "met": 13, "phe": 14, "pro": 15,
 "ser": 16, "thr": 17, "trp": 18, "tyr": 19,
 "val": 20, "Ter": 21}

*def* nuc(site1):
 feature1 = *sum*(*map*(*int*, re.findall("\d+", site1)))
 feature2 = site1[site1.index(">") - 1]
 feature3 = site1[site1.index(">") + 1]

feature2_ = c_feature[feature2]

feature3_ = c_feature[feature3]
 *return* [feature1, feature2_, feature3_]

*def* aa(site1):
 feature1 = *int*(re.findall("\d+", site1)[0])
 feature1_idx = site1.index(*str*(feature1))
 feature2 = site1[feature1_idx - 3:feature1_idx].lower()
 feature3 = site1[feature1_idx + 3:feature1_idx + 6].lower()
 feature2_ = aa_feature[feature2]
 feature3_ = aa_feature[feature3]
 *return* [feature1, feature2_, feature3_]

*def* exon_intron(ex):
 *if* "exon" *in* ex.lower():
 *return int*(ex[4:])
 *if* "intron" *in* ex.lower():
 *return int*(ex[6:]) + 10

**Supplementary Note 2**

**Encode graph feature method**

We extract 3 distance features that calculated the minimum distances to three hub-node, including the minimum distance to the cPKU hub node, minimum distance to the mPKU hub node and minimum distance to the MHP hub node. The 5 attributions of a node include Degree, Edge, betweenness, Page rank, Closeness, and Eccentricity. The detailed encode framework and core of code about encode methods are as follow:

**
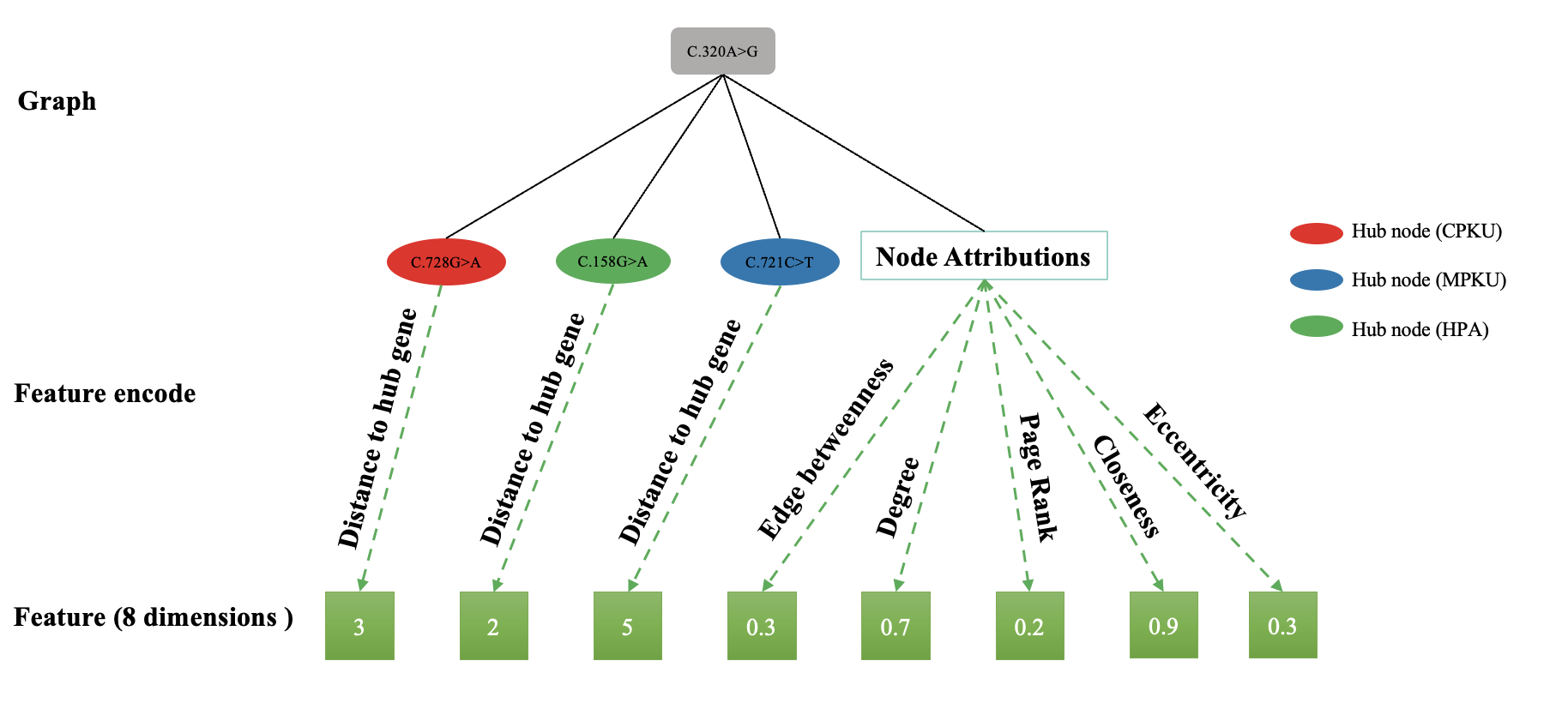
**

**Code Snippet**

*def* graph_feature(g,node):
 deg = g.degree()
 bet = g.edge_betweenness()
 page = g.pagerank()
 clo = g.closeness()
 eccen = g.eccentricity()
 feature = [deg[node],bet[node],page[node],clo[node],eccen[node],
 *len*(g.get_shortest_paths(node,'c.728G>A')[0]),
 *len*(g.get_shortest_paths(node,'c.158G>A')[0]),
 *len*(g.get_shortest_paths(node,'c.721C>T')[0])]
 *return* feature
