# Supplementary figures and images for: Allelic phenotype prediction of phenylketonuria based on the machine learning method
Source: Hum Genomics. 2023 Mar 31;17:34. doi: 10.1186/s40246-023-00481-9 (PMC10064562; doi:10.1186/s40246-023-00481-9)

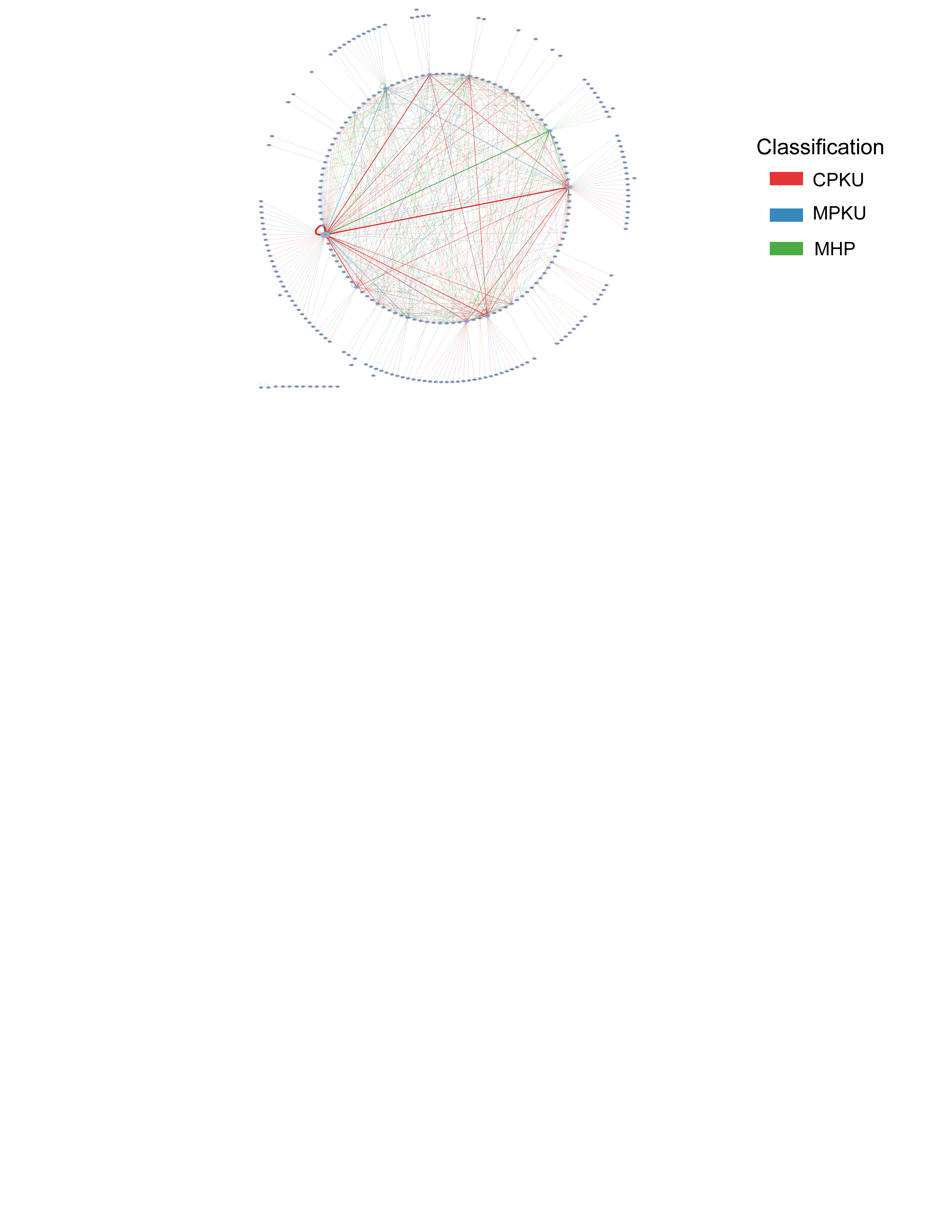

Supplement: Supplementary file 6 — Additional file 6: Fig. S4. Connectivity of allelic mutations in the PAH gene. [file 40246_2023_481_MOESM6_ESM.tif]

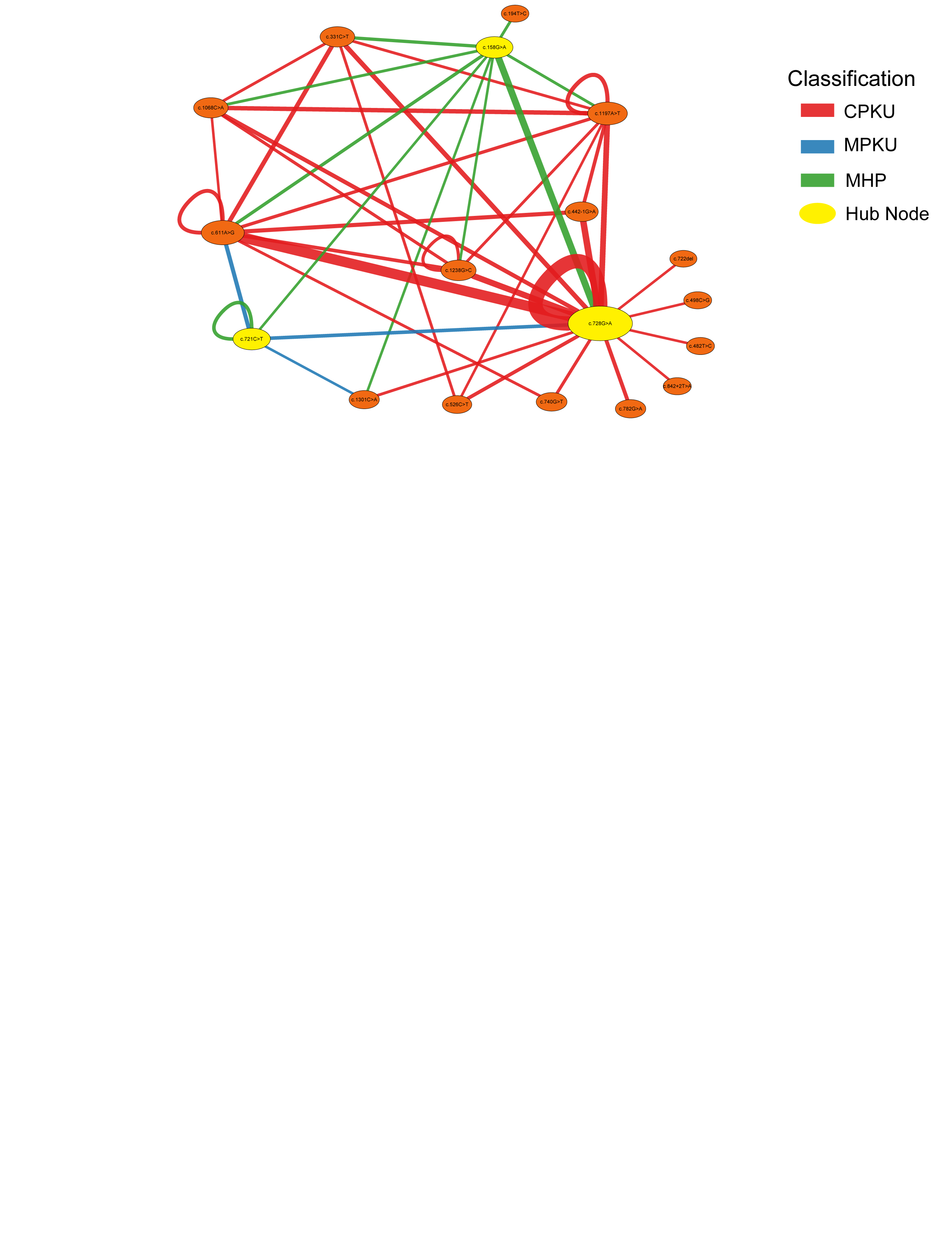

Supplement: Supplementary file 7 — Additional file 7: Fig. S5. Hub nodes for cPKU, mPKU and MPH. [file 40246_2023_481_MOESM7_ESM.tif]

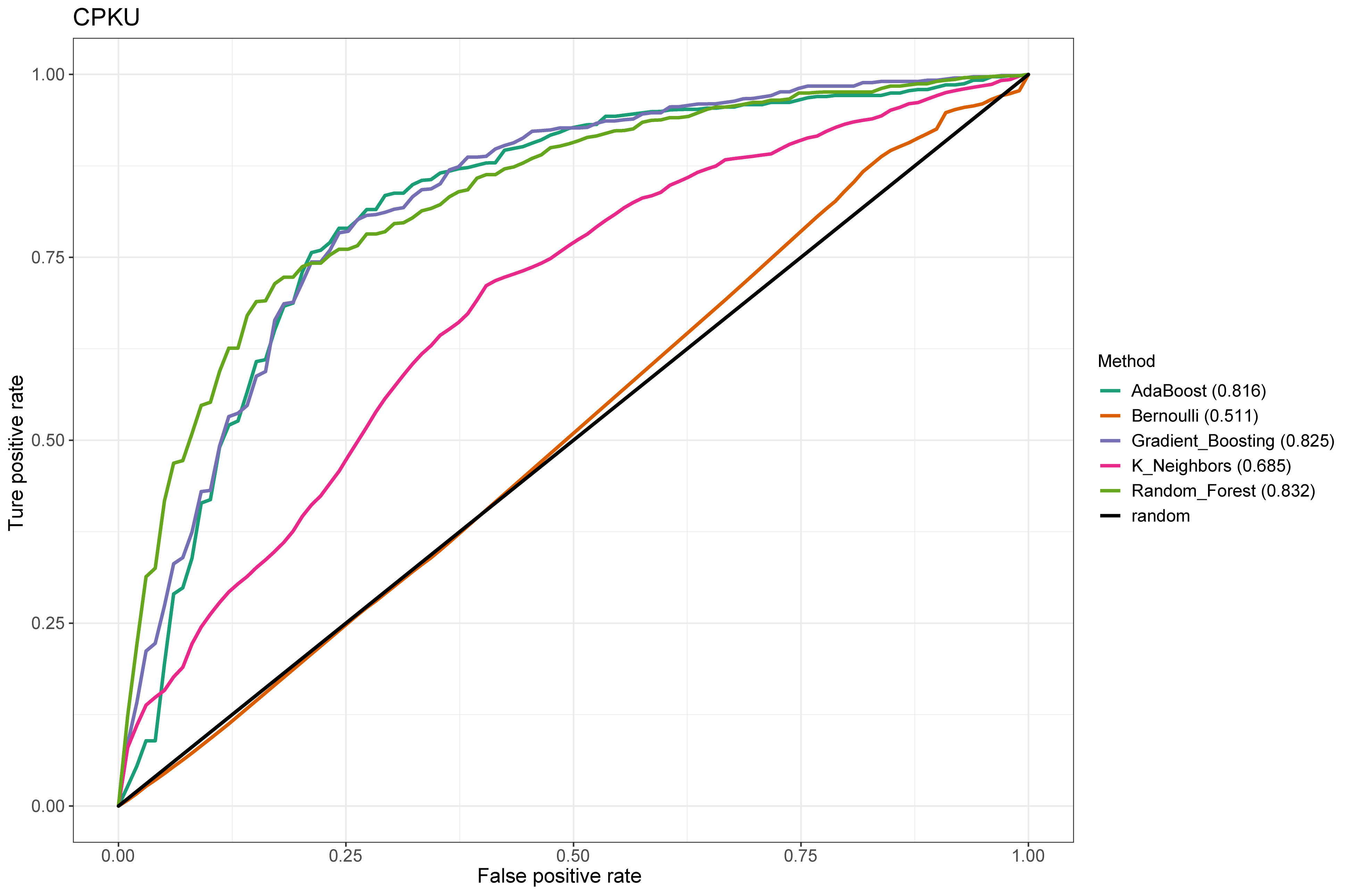

Supplement: Supplementary file 10 — Additional file 10. Figure S6. Compared the RF method with other classification methods. [file 40246_2023_481_MOESM10_ESM.tif]
